# Long-reach 60-GHz MMWoF link with free-running laser diodes beating

**DOI:** 10.1038/s41598-018-32058-1

**Published:** 2018-09-12

**Authors:** Cheng-Ting Tsai, Chien-Cheng Li, Chi-Hsiang Lin, Chun-Ting Lin, Sien Chi, Gong-Ru Lin

**Affiliations:** 1 Graduate Institute of Photonics and Optoelectronics, Department of Electrical Engineering, National Taiwan University, Taipei, 10617 Taiwan, ROC; 2 Institute of Photonic System, National Chiao-Tung University, Tainan 711, Taiwan, ROC; 3 Institute of Electro-Optical Engineering and the Institute of Photonic System, National Chiao Tung University, Hsinchu City 300, Taiwan, ROC

## Abstract

With the remote beating of two mutually incoherent laser carriers, the local-oscillator-free long-reach millimeter-wave over fiber (MMWoF) link at 60-GHz band is demonstrated. The unique schemes of the proposed MMWoF are the wavelength-locked colorless laser diode (CLD) modulator, the mutually incoherent optical carrier for heterodyne MMW generation, and the square-law power envelope detection at receiving end. By directly encoding the single-mode with the CLD modulator, the single-carrier modulated QAM-OFDM data is achieved to release the RF power fading after fiber transmission. The mutually incoherent laser beating enables the optical heterodyne MMW generation with two independent optical carriers, which provides the advantages of local-oscillator-free operation and rules out the requirement of dual-mode optical carrier delivery from central office. At the wireless receiving end, the received QAM-OFDM data is self-down-converted to the baseband by employing the square-law power envelope detection. This eliminates the requirement of local oscillator and rules out the influence of the MMW carrier frequency fluctuation between two mutually incoherent lasers (used at central office and remote node), which effectively provides the MMW carrier immunity against the down-conversion instability caused by clock jitter or carrier incoherence. This architecture ensures the transmission of 16.5-Gbit/s 32-QAM OFDM data over 50 km in SMF and 3 m in free-space with the FEC certificated error vector magnitude of 12%, signal-to-noise ratio (SNR) of 18.4 dB, and bit error rate of 3.8 × 10^−3^. For multi-channel DWDM-PON applications, the proposed local-oscillator-free MMWoF link can successfully perform 11 DWDM channels of 32-QAM OFDM data access at 16.5 Gbit/s per channel via the wavelength controlling of the CLD modulation stage and the detuning of the beating carrier at remote node.

## Introduction

Nowadays, the 5th generation (5 G) wireless mobile networks with the highly adaptive access capability have been comprehensively investigated to take over the current 4 G mobile networks in near future, which can support the real-time multimedia services at 100 M-1 Gbit/s for metropolitan areas with enhanced spectral efficiency, reduced latency, and improved network coverage for mobile users^1,2^. To avoid the currently used frequency slots at ultra-high frequency (UHF) and super-high frequency (SHF) bands, the 5 G mobile network infrastructures are planning to implement by exploring the wide-bandwidth and license-free frequency slots in the currently available millimeter wave (MMW) region^3–5^. In 2014, the MMW carriers at 28 and 38 GHz were considered to experimentally demonstrate the high-speed 5 G mobile communications with available bandwidth of >1 GHz^6,7^. At present stage, the Federal Communications Commission has officially announced the available frequency slots in the MMW bands for 5 G mobile networks at 28, 37, 39, and 64–71 GHz^8^. However, even if there are available MMW frequency bands for 5 G mobile networks, high atmospheric attenuation during free-space MMW transmission still needs to be considered^9,10^. To spread the allowable range of coverage for the MMW carriers, the fusion of the MMW wireless access links with currently established optical fiber networks for future 5 G mobile service is a potential solution so-called MMW over fiber (MMWoF) system^11–13^, which not only delivers the MMW carriers from central office (CO) to 5 G mobile microcells via active optical networks (AONs)^14^, but also constructs the wired connections between MMW base stations (BSs) and the passive optical network (PON)^15^. Typically, the method to obtain the MMW carrier in the MMWoF system is the optical heterodyne between the received optoelectrically converted data-stream and a local oscillator at receiving end^16^. Unfortunately, such an architecture suffers from the construction cost with local oscillator and related MMW components. The photomixing is a simple and cost-effective technique for MMW generation, which uses the dual-mode optical carrier to transmit the data from CO and employs the optical heterodyne for optical-to-MMW carrier conversion after receiving at remote node to simplify the infrastructure of the MMW BSs^17–19^.

To synthesize the typical dual-mode optical carrier for the optical heterodyne MMWoF systems, the central-carrier suppressed double-sideband (CCS-DSB) optical carrier with high coherence feature is usually employed as representative candidate, which can be generated by externally modulating a continuous-wave (CW) laser with a nully-biased Mach-Zehnder modulator (MZM)^20,21^. The data-stream can be encoded onto the dual-mode optical carrier via the external modulation with an additional optical modulator^22,23^; however, such an architecture relies on electro-optic double-sideband data modulation and suffers from chromatic dispersion induced power fading after fiber transmission^24,25^. This severely limits the allowable transmission distance of the MMWoF even with single-mode fiber (SMF)^14^. Technically, the RF power fading induced modulation spectral notch can be cost-ineffectively released by using optical single-sideband modulation with dual-electrode MZM^26–29^. Alternatively, the directly modulated laser with dual wavelengths can also be considered to provide the either single-mode or dual-mode optical carrier modulation with enhanced modulation bandwidth and suppressed intensity noise under injection^30,31^. Particularly, similar technology treats the slave laser as both modulator and amplifier that has ever been reported. For the MMWoF with embedding dual carriers, the wavelength-locking a single-mode slave laser with the dual-mode master has also emerged to achieve optically single-sideband modulation to entirely release the induced RF power fading effect;^32^ however, the low injection efficiency and narrow wavelength tolerance of conventional laser diode restricts its applicability for multi-channel universal wavelength-division-multiplexed passive optical network (DWDM-PON) applications.

In view of versatile approaches for dual wavelength applications, directly combining two independent CW lasers is the simplest way to generate the mutually incoherent dual-mode optical carrier, as which can arbitrarily adjust its mode spacing and wavelength by detuning either one^33^. Nevertheless, the currently proposed incoherent dual-mode carrier still requires an external modulator to encode the data. Besides, one serious drawback of this technology is that the mutual incoherence between two independent lasers also fluctuates the frequency of the beat MMW carrier after optical heterodyne detection at remote node. Such an approach suffers from the down-conversion instability to make it inappropriate for most MMWoF systems. More recently, the microwave power detector with the square-law power envelope detection function has been proposed to considerably release the frequency instability during down-converted mixing process, which allows the MMW signal self-converting down to the baseband without using local oscillator at remote node^12,34^.

In this work, the mutually incoherent optical carriers with two wavelengths delivered by two independent lasers are employed to establish the local-oscillator-free long-reach MMWoF link at wireless frequency of 60 GHz. The MMW carrier is synthesized by remotely combining the down-stream single-carrier transmitted data with another independent single-mode carrier at the optical receiving end. The mutual coherence between these two free-running laser carriers is almost zero such that the phase/frequency fluctuation of their beat MMW carrier is extremely large without any control. The direct modulation of down-stream data is performed by using a wavelength-locked colorless laser diode (CLD) modulator, which allows the single-mode optical carrier to carry the quadrature amplitude modulation (QAM) and orthogonal frequency division multiplexing (OFDM) data. As the incoherence between two optical carriers from CO and remote node inevitably fluctuates the optically heterodyned MMW carrier frequency, it induces tremendous down-conversion frequency instability at wireless receiving end. This issue is released by employing the square-law power envelope detection technique, which makes the MMW central carrier and its carried QAM-OFDM data self-down-convert to baseband without disregarding the carrier frequency fluctuation. To flatten the signal-to-noise ratio (SNR) of the received QAM-OFDM data, the pre-emphasis technique is introduced before encoding onto the CLD modulator. The allowable modulation bandwidth of the optimized QAM-OFDM data is analyzed before and after 50-km SMF transmission. After remotely beating the mutually incoherent two-wavelength optical carriers at optical receiver, the MMW wireless transmission performances of the 3-m free-space transmitted QAM-OFDM data are examined, including the error vector magnitude (EVM), SNR and bit error rate (BER). To confirm the multi-channel DWDM flexibility, the allowable channel number availably offered by such a mutually incoherent dual-wavelength optical carriers for the MMWoF link is surveyed.

## Results

### Theoretical principle: a. photonic 60-GHz MMW carrier beating of two mutually incoherent optical carriers. b. square-law power envelope detection of 60-GHz MMW QAM-OFDM data

**Figure 1:**
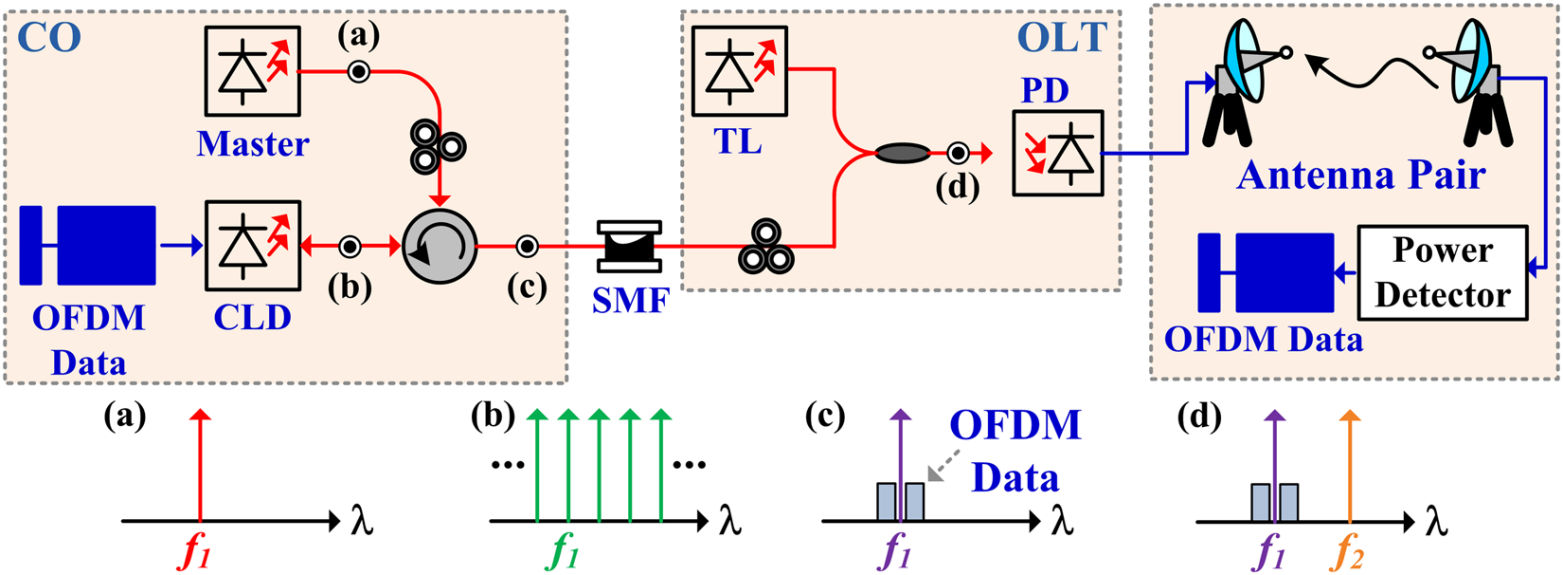
Concept of photonic MMW carrier beating with two mutually incoherent optical carriers for MMWoF system.

The concept of the mutually incoherent dual-wavelength optical carrier generation and the remotely optical heterodyne of the MMW carrier synthesis in the MMWoF system is illustrated in Fig. 1. This architecture consists of key techniques, including: (i) the single-carrier modulation via the wavelength-locked CLD and (ii) the square-law power envelope detection of the mutually incoherent dual-wavelength optical carriers heterodyned MMW QAM-OFDM data. In the CO, a free-running tunable laser (TL) (Fig. 1(a)) is employed as the optical master for implementing optical baseband transmission. For QAM-OFDM encoding onto a specific DWDM channel, a directly modulated slave CLD is employed as the optical modulator and mode-selective amplifier (Fig. 1(b)) via the master controlled wavelength-locking (Fig. 1(c)).

After the wavelength-locking via master injection, the electrical field of the carrier with QAM-OFDM data output from the CLD modulator is expressed as^35,36^

1 $${E}_{OFDM}(z,t)=[\sqrt{{P}_{0}+\sum _{n=0}^{N}{P}_{n}\,\cos (2\pi n{f}_{subcarrier}t+{\theta }_{n})}]\cos [kz-2\pi ({f}_{1}+\Delta {f}_{1})t],={E}_{OFDM}(t)\cos [kz-2\pi ({f}_{1}+\Delta {f}_{1})t]$$

where *P*_0_ denotes the average power of the CLD, *N* the total OFDM subcarrier number, *k* the propagation constant, *f*_1_ the optical frequency, and Δ*f*_1_ the inherent frequency fluctuation caused by the CLD, and *P*_*n*_, *f*_*subcarrier*_, and *θ*_*n*_ denote the optical power, the frequency, and the phase of the *n*th OFDM subcarrier, respectively. After SMF transmission, the mutually incoherent dual-wavelength optical carriers is obtained by combining the down-stream transmitted CLD carrier with another localized TL provided carrier in an optical network unit (ONU). Such a free-running and coherence independent dual-wavelength optical carriers exhibits only optical single-carrier modulation to completely release the chromatic dispersion induced RF power fading effect after optical heterodyne detection (Fig. 1(d)). Assuming that the electrical field of the incoherent dual-mode optical carrier is expressed as

2 $$\mathop{{E}_{DM}}\limits^{\rightharpoonup }(z,\,t)={E}_{OFDM}(t)\cos [kz-2\pi ({f}_{1}+\Delta {f}_{1})t]+{E}_{TL,local}\,\cos [kz-2\pi ({f}_{2}+\Delta {f}_{2})t],$$

where *E*_*TL, local*_ denotes the amplitude, *f*_2_ the optical frequency, and Δ*f*_2_ the frequency fluctuation of the localized TL. Note that the frequency spacing between *f*_1_ and *f*_2_ is set as 60 GHz for the optically heterodyned MMW carrier. After optical heterodyne conversion, the photocurrent converted from the received mutually incoherent optical carriers at the PD is expressed as

3 $${i}_{PD}=R\cdot {\{{E}_{OFDM}(t)\cos [kz-2\pi ({f}_{1}+{\rm{\Delta }}{f}_{1})t]+{E}_{TL,local}\cos [kz-2\pi ({f}_{2}-{\rm{\Delta }}{f}_{2})t]\}}^{2}=R\cdot \{\begin{array}{l}{E}_{OFDM}^{2}(t)\frac{1+\,\cos \,2[2\pi ({f}_{1}-{\rm{\Delta }}{f}_{1})t]}{2}+{E}_{TL,\,local}^{2}\frac{1+\,\cos \,2[2\pi ({f}_{2}-{\rm{\Delta }}{f}_{2})t]}{2}\\ +{E}_{OFDM}(t){E}_{TL,local}\,\cos [2\pi ({f}_{1}+{f}_{2}+{\rm{\Delta }}{f}_{1}-{\rm{\Delta }}{f}_{2})t]\\ +{E}_{OFDM}(t){E}_{TL,local}\,\cos [2\pi ({f}_{1}-{f}_{2}+{\rm{\Delta }}{f}_{1}+{\rm{\Delta }}{f}_{2})t]\end{array}\},$$

where *R* denotes the responsivity of the PD. In Eq. 3, the fourth terms at the right-hand side is the MMW central carrier with the delivered QAM-OFDM data. Typically, the MMW carried data at the wireless receiving end must be frequency down-converted to the baseband by mixing a microwave local-oscillator. Owing to the phase irrelevance and frequency unsynchronization between the mutually incoherent dual-wavelength optical carriers, the summed frequency fluctuation of the optically heterodyned MMW central carrier (Δ*f* = Δ*f*_1_ + Δ*f*_2_) seriously make the down-conversion instability. To avoid such a noise variation originated from beating the free-running and coherence-independent carriers, the square-law power envelope detector is employed, which can self-down-convert the QAM-OFDM data from MMW band to the baseband without using microwave local-oscillator and rules out the down-conversion instability induced noise term. At the wireless receiving end, the electrical power of the received OFDM data after passing through the square-law power envelope detector is expressed as

4 $${P}_{Detected}(t)={R}^{2}\cdot {\{{E}_{OFDM}(t){E}_{TL,local}\cos [2\pi ({f}_{1}-{f}_{2}+{\rm{\Delta }}f)t]\}}^{2}={R}^{2}{E}_{OFDM}^{2}(t){E}_{TL,\,local}^{2}{\cos }^{2}[2\pi ({f}_{1}-{f}_{2}+{\rm{\Delta }}f)t]={P}_{OFDM}(t)\{\cos [2\pi (2{f}_{1}-2{f}_{2}+2{\rm{\Delta }}f)t]+1\}={P}_{OFDM}(t)+{P}_{OFDM}(t)\cos [2\pi (2{f}_{1}-2{f}_{2}+2{\rm{\Delta }}f)t],$$

In Eq. 4, the first term at the right-hand side denotes the electrical power of the down-converted OFDM data, which clearly indicates the banishment of down-conversion induced frequency fluctuation on the OFDM data. On the other hand, the obtained electrical power of the down-converted OFDM data is constituted by multiplying the optical power of the OFDM data with the square of the responsivity and the optical power of the localized TL. Based on such a relationship, the electrical power of the down-converted OFDM data can be effectively boosted by increasing the optical power of the localized TL at a fixed responsivity of the PD receiver. In the proposed concept, the localized TL is thus treated as the key light source to not only provide a remotely coherence-independent optical carrier but also amplify the receiving power of the down-converted OFDM data.

### Throughput characteristics of wavelength-locked CLD modulator and Encoding the 64-QAM OFDM Data via the wavelength-locked CLD modulator for optical down-stream transmission

**Figure 2:**
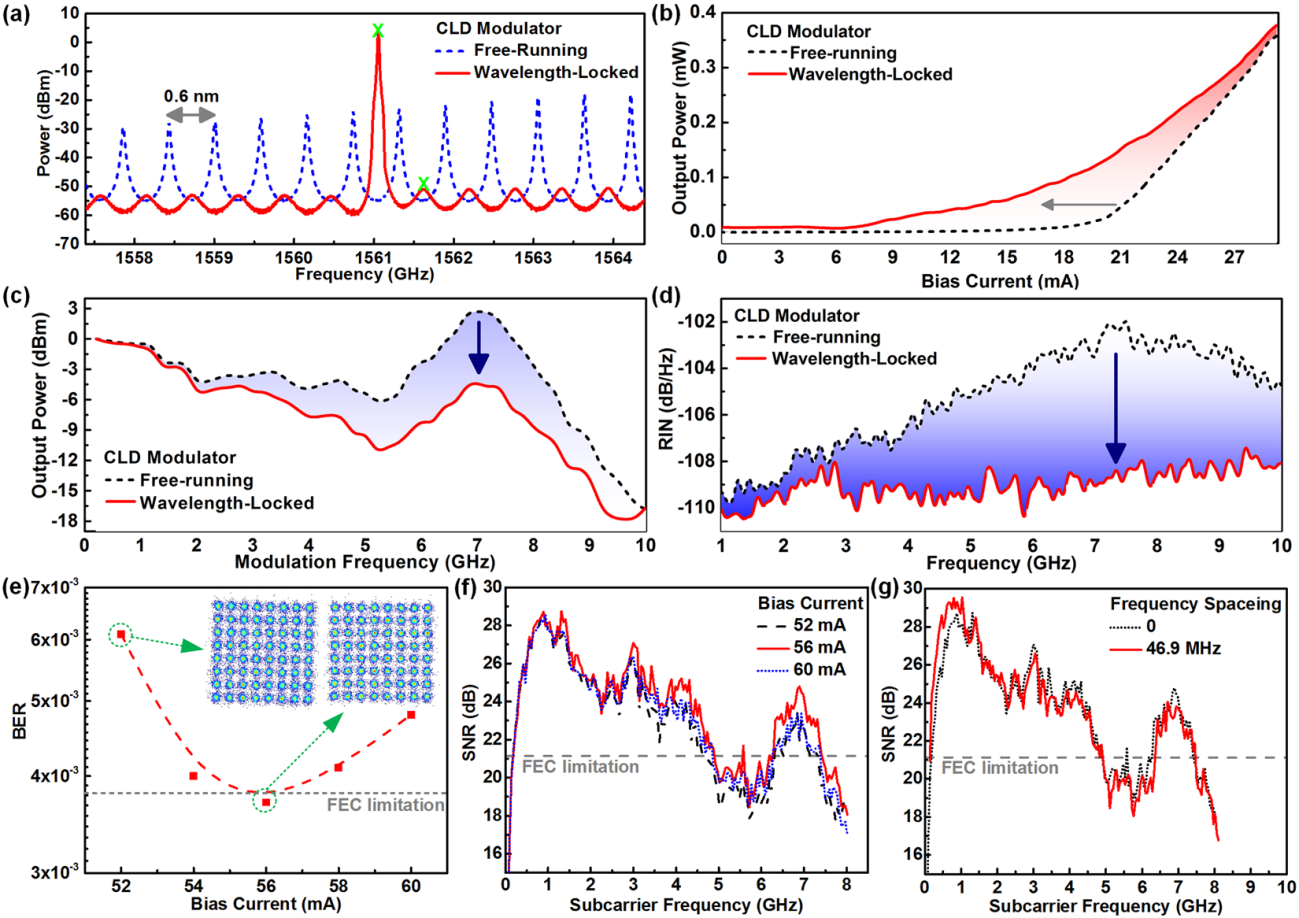
Output characteristics of wavelength-locked CLD and transmission performance of carried 64-QAM OFDM data at optical BtB transmission. (**a**) Optical spectra, (**b**) power-to-current responses, (**c**) frequency responses, and (**d**) RIN responses of CLD modulator with and without wavelength-locking. (**e**) BER and constellation plots. (**f**) SNR responses at different bias currents. (**g**) SNR responses with different frequency spacing deviated from the DC point.

Externally seeding the CLD modulator with master not only controls the selected DWDM channel wavelength but also enlarges the modulation bandwidth with extremely low intensity noise during the QAM-OFDM data encoding. Figure 2(a) shows the optical spectrum of the CLD modulator at a bias current of 56 mA before and after the 3-dBm external injection. The free-running CLD modulator with increased cavity length and decreased front-facet reflectance can provide dense longitudinal modes with wavelength spacing of 0.6 nm. The master injection at 3 dBm enforces the CLD modulator to select single longitudinal mode with a side mode suppression ratio (SMSR) as high as 47.8 dB (indicated with the labels “X” in Fig. 2(a)). Note that the red-shifted longitudinal mode is due to the inherent stable locking of the CLD is located at a slightly longer wavelength owing to the effective threshold reduction^37^. As shown in Fig. 2(b), the power-to-current response demonstrates that the threshold current of the slave CLD modulator is reduced from 19 to 9 mA after external master injection, which also raises the output power of the slave CLD modulator at a fixed bias point. Figure 2(c) illustrates the modulation frequency responses of the CLD modulator with and without the wavelength-locking by master. At the free-running condition, the CLD modulator provides a 6-dB modulation bandwidth of 8.4 GHz for carrying the QAM-OFDM data, which slightly shrinks to 7.5 GHz after wavelength-locking with master, as the roll-off effect declines the power-to-frequency throughput response^30,31^. That is, either the large bias or the strong injection substantially up-shifts the relaxation oscillation frequency of the CLD modulator, which therefore sacrifices the low frequency energy to extend the broadband response. On the other hand, wavelength-locking the CLD modulator not only increases its relaxation oscillation frequency but also suppresses its relative intensity noise (RIN)^38^. As shown in Fig. 2(d), the RIN peak of the CLD modulator can be up-shifted from 7.4 to >10 GHz after intense master injection, which greatly suppresses the RIN level from −102.2 to −108.2 dB/GHz. Although the master wavelength-locking shrinks the 6-dB bandwidth of the slave CLD modulator by up-shifting the relaxation oscillation frequency, the raised output power and reduced RIN still provide excellent modulation features to favor the QAM-OFDM data encoding.

As the average power of the electrical 64-QAM OFDM data is fixed at 1.6 dBm, the related bias point of the wavelength-locked CLD modulator must be optimized to prevent the QAM-OFDM waveform clipping in time domain by direct modulation below threshold (I_bias_-I_th_ < I_OFDM_) or beyond saturation (I_sat_-I_bias_ < I_OFDM_). Figure 2(e) shows the BERs and the related constellation plots of the optical back-to-back (BtB) transmitted 64-QAM OFDM data at the OFDM bandwidth of 8 GHz carried by the wavelength-locked CLD modulator as a function of bias currents. The delivered QAM-OFDM data optimizes its EVM to 7.1% and BER to 3.7 × 10^−3^ by increasing the DC bias current of the CLD modulator to 56 mA, which results from the reduced RIN and suppressed waveform clipping after optimization. Overly biasing the CLD modulator beyond 60 mA may cause the waveform distortion at upper part to degrade the EVM and BER of the QAM-OFDM data to 7.5% and 4.8 × 10^−3^, respectively. This is caused by either the saturation clipped or the roll-off declined throughput response of the CLD modulator, which degrades the SNR response of the QAM-OFDM data carried by OFDM subcarriers at high frequencies. Figure 2(f) shows the SNR responses of the QAM-OFDM data carried by wavelength-locked CLD modulator biased at different currents. With increasing the bias current of the CLD modulator to 56 mA, the SNR response of the carried QAM-OFDM data can be increased by >2 dB (from 22.1 to 23 dB), whereas the over bias inevitably degrades the average SNR to a level comparable with the insufficient bias cases. To prevent the SNR degradation of the 64-QAM OFDM data at low OFDM subcarrier frequencies by the cut-off response of the electrical components, the first OFDM subcarrier frequency is enlarged to 46.9 MHz away from the DC point. As shown in Fig. 2(g), the SNR response of the 64-QAM OFDM data at low frequencies can be improved by >6 dB after enlarging the starting frequency spacing for allocating the OFDM subcarriers to 46.9 MHz, which optimizes the EVM to 7%, average SNR to 23.1 dB, and BER to 3.4 × 10^−3^.

### Improving the carried 64-QAM OFDM data with pre-emphasis technology

**Figure 3:**
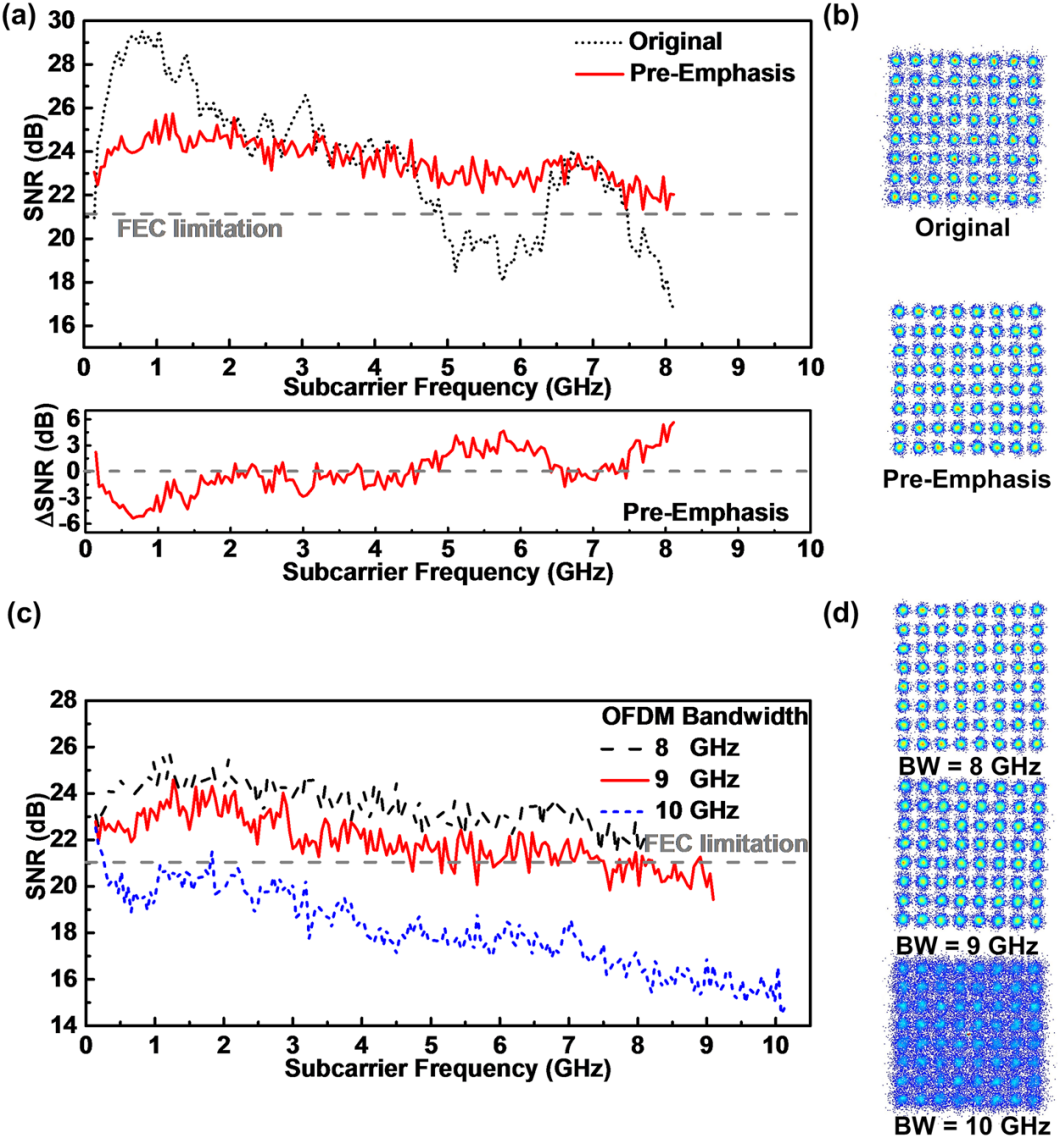
Transmission performance of 64-QAM OFDM data with and without pre-emphasis. (**a**) SNR responses and difference, and (**b**) constellation plots of 64-QAM OFDM data with and without pre-emphasis. (**c**) SNR responses and (**d**) constellation plots of the 64-QAM OFDM data at different modulation bandwidths after pre-emphasis.

To further improve the 64-QAM OFDM data carried by the wavelength-locked CLD modulator, the pre-emphasis technology is employed to flatten the SNR response over the entire encoding bandwidth of the received QAN-OFDM data. The upper of Fig. 3(a) shows the SNR responses of the QAM-OFDM data carried by the wavelength-locked CLD modulator with and without the pre-emphasis technology. The average SNR of the QAM-OFDM data can be improved to 23.5 dB after implementing the pre-emphasis, which meets the FEC required SNR criterion of 21.1 dB (providing the BER criterion of 3.8 × 10^−3^). The lower of Fig. 3(a) illustrates the SNR improvement after using the pre-emphasis. Figure 3(b) illustrates the constellation plots of the received QAM-OFDM data with and without the pre-emphasis, in which the EVM can be improved to 6.6% after pre-emphasis. By implementing the emphasis, the allowable bandwidth of the 64-QAM OFDM data carried by the wavelength-locked CLD modulator can also be enlarged as well. Figure 3(c) shows the SNR responses of the optical BtB transmitted QAM-OFDM data at different modulation bandwidths.

**Figure 4:**
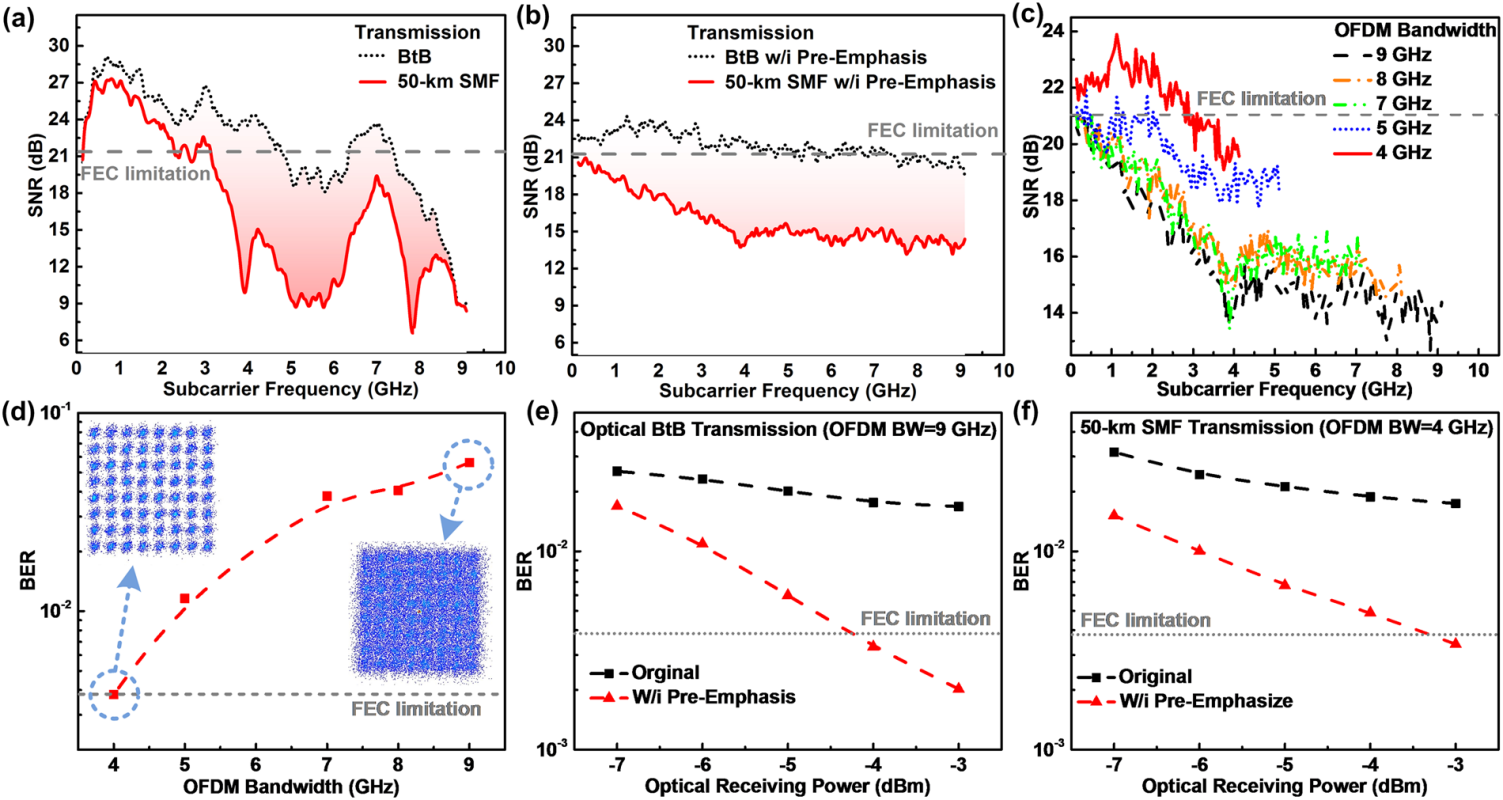
Performance of 64-QAM OFDM data at the modulation bandwidth of 9 GHz before and after 50-km SMF transmission. SNR responses of 64-QAM OFDM data before and after 50-km SMF transmission (**a**) without and (**b**) with pre-emphasis. (**c**) SNR responses, (**d**) BERs and related constellation plots of 50-km SMF transmitted 64-QAM OFDM data with its modulation ranging from 4 to 9 GHz. BER versus receiving power of 64-QAM OFDM data at a modulation bandwidth of 9 and 4 GHz (**e**) before and (**f**) after 50-km SMF transmission.

Enlarging the modulation bandwidth to 9 GHz effectively reduces the overall SNR response by 1 dB, which is attributed to the energy redistribution over the extended bandwidth such that the pre-emphasis consumes the energy for flattening the SNR response. This degrades the average SNR and BER of the QAM-OFDM data from 23.5 to 21.9 dB and from 5.2 × 10^−4^ to 2.7 × 10^−3^, respectively. By further increasing the modulation bandwidth up to 10 GHz, the SNR response of the QAM-OFDM data seriously decreases by >2.5 dB, as the CLD modulator is no longer available for providing sufficient energy to perform the pre-emphasis. This degrades the average SNR and BER of the received QAM-OFDM data to 17.9 dB and 2.8 × 10^−2^, respectively, which fails to meet the FEC criterion. Figure 3(d) illustrates the constellation plots of the received QAM-OFDM data with the pre-emphasis at different modulation bandwidths, which reveals an EVMs of 6.6%, 8%, and 12.7% for a modulation bandwidths of 8, 9, and 10 GHz, respectively. Note that both 8- and 9-GHz wideband QAM-OFDM data can pass the FEC criterion, whereas the 10-GHz wideband QAM-OFDM fails to be successfully decoded even with aforementioned processing. To evaluate the long-reach performance at an optical receiving power of −3 dBm, the SNR responses of the received 64-QAM OFDM data at the modulation bandwidth of 9 GHz before and after 50-km SMF transmission are compared in Fig. 4(a). After passing through the 50-km SMF, the chromatic dispersion induced RF power fading seriously decreases the SNR of the received QAM-OFDM data at high frequencies with the largest degradation of >9 dB, which inevitably reduces the SNR from 20.7 to 14.6 dB in average. Apparently, Fig. 4(b) illustrates the SNR response of the received QAM-OFDM data before and after 50-km SMF transmission. Even with the pre-emphasis for flattening the BtB transmitted SNR response, the long-reach SNR of the QAM-OFDM data still fails to meet the FEC criterion as the enhancement by pre-emphasis dose not overcome the power fading induced degradation on receiving power.

To meet the FEC criterion, the allowable modulation bandwidth of the long-reach 50-km SMF transmitted QAM-OFDM data with the pre-emphasis has to be educed accordingly. As shown in Fig. 4(c,d), shrinking the modulation bandwidth from 9 to 4 GHz retrieves the average SNR of the transmitted QAM-OFDM data from 15.7 to 21.6 dB to concurrently optimize the BER and EVM from 5.6 × 10^−2^ to 3.8 × 10^−3^ and 16.4% to 8.3%, respectively, for meeting the FEC criterion. In addition, Fig. 4(e,f) show the corresponding BER responses versus optical receiving power. Under optical BtB transmission, the lowest BER of 1.6 × 10^−2^ is obtained for the 9-GHz QAM-OFDM data at an optical receiving power of −3 dBm, which can be further improved to 2 × 10^−3^ after implementing the pre-emphasis. The BER increases beyond the FEC limitation after reducing the optical receiving power to <−4 dBm. After long-reach transmission in 50-km SMF, the lowest BER of 3.4 × 10^−3^ can be obtained at receiving power of −3 dBm for the 4-GHz QAM-OFDM data. In comparison with optical BtB transmission at 9-GHz data bandwidth, the 50-km SMF based long-reach BER is degraded with a receiving power penalty of 1 dB owing to the RF power fading effect. Even with the pre-emphasis technology for flattening the SNR response, the QAM-OFDM data still requires more power to maintain its SNR during long-reach transmission. Besides, the slope of BER vs. receiving power show that the BER performance of the 50-km SMF transmitted QAM-OFDM data with pre-emphasis becomes less sensitive to the optical receiving power after 50-km SMF transmission.

### Wireless 16-QAM OFDM performance after converting the carrier from optical to MMW at 60 GHz

**Figure 5:**
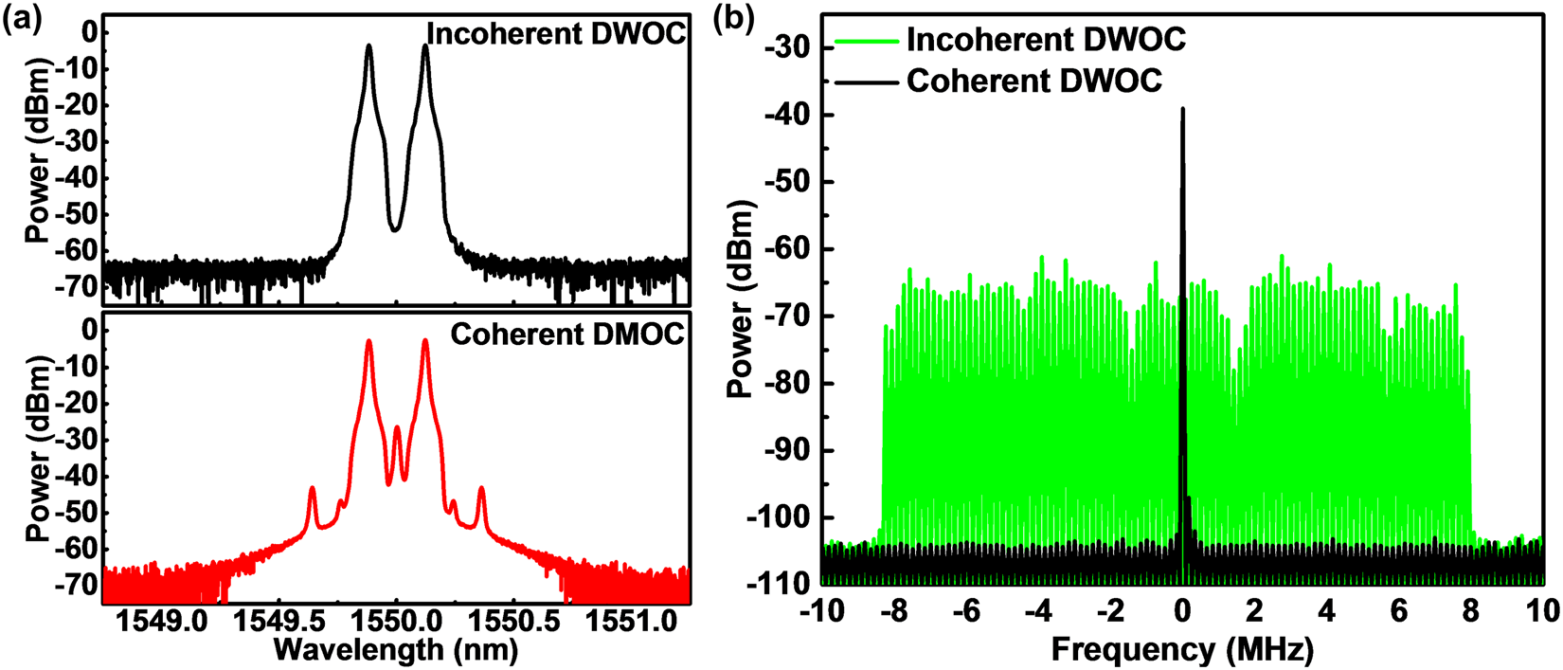
Carrier frequency fluctuation of 60-GHz MMW signal optically heterodyned from mutually incoherent and coherent dual-wavelength optical carriers. (**a**) Optical spectra, and (**b**) beat electrical spectra of the incoherent and coherent dual-wavelength laser sources for optical heterodyne of MMW central carriers. DWOC: dual-wavelength optical carriers.

At beginning, the carrier frequency fluctuation of the 60-GHz MMW signal optically heterodyned from mutually incoherent dual-wavelength optical carriers is analyzed and compared with that obtained by using a standard CCS-DSB modulation technology. As a result, Fig. 5(a) illustrates the optical spectra and Fig. 5(b) compares the electrical spectra of the incoherent and coherent dual-mode optical carriers and their optically heterodyned MMW carriers at 60 GHz. The frequency of the incoherently beat MMW carrier is seriously fluctuated to randomly scan across its central peak over a spectral linewidth of 16.2 MHz. The frequency fluctuation mainly occurs from the relative wavelength variation between two independent laser diodes. As the injection locking mechanism forces the slave colorless laser diode to concurrently lock its wavelengths with two independent laser diodes, the relative frequency fluctuation between two master laser diodes still exists such that the optically heterodyned MMW carrier exhibits corresponding frequency variation. In contract, the coherently generated dual-wavelength optical carriers can beat a very stabilized MMW carrier with its spectral linewidth only spreading over 81.4 kHz, because of the high coherence feature of the completely coherent CCS-DSB carrier pair. Even through the incoherent dual-mode optical carriers beat a broadband fluctuated and scanned MMW carrier, our work will demonstrate that the 60-GHz MMW carried QAM-OFDM data can be stably down-converted to the baseband with high immunity to the carrier frequency instability by using the power detector with the square-law power envelope detection. To suppress additional noise in the proposed MMWoF link, a microwave bandpass filter with a passband linewidth of 10 GHz at central frequency of 60 GHz is employed to reduce amplifier noises before down-conversion, which somewhat limits the allowable modulation bandwidth within 5 GHz for the MMW carried 32-QAM OFDM data.

**Figure 6:**
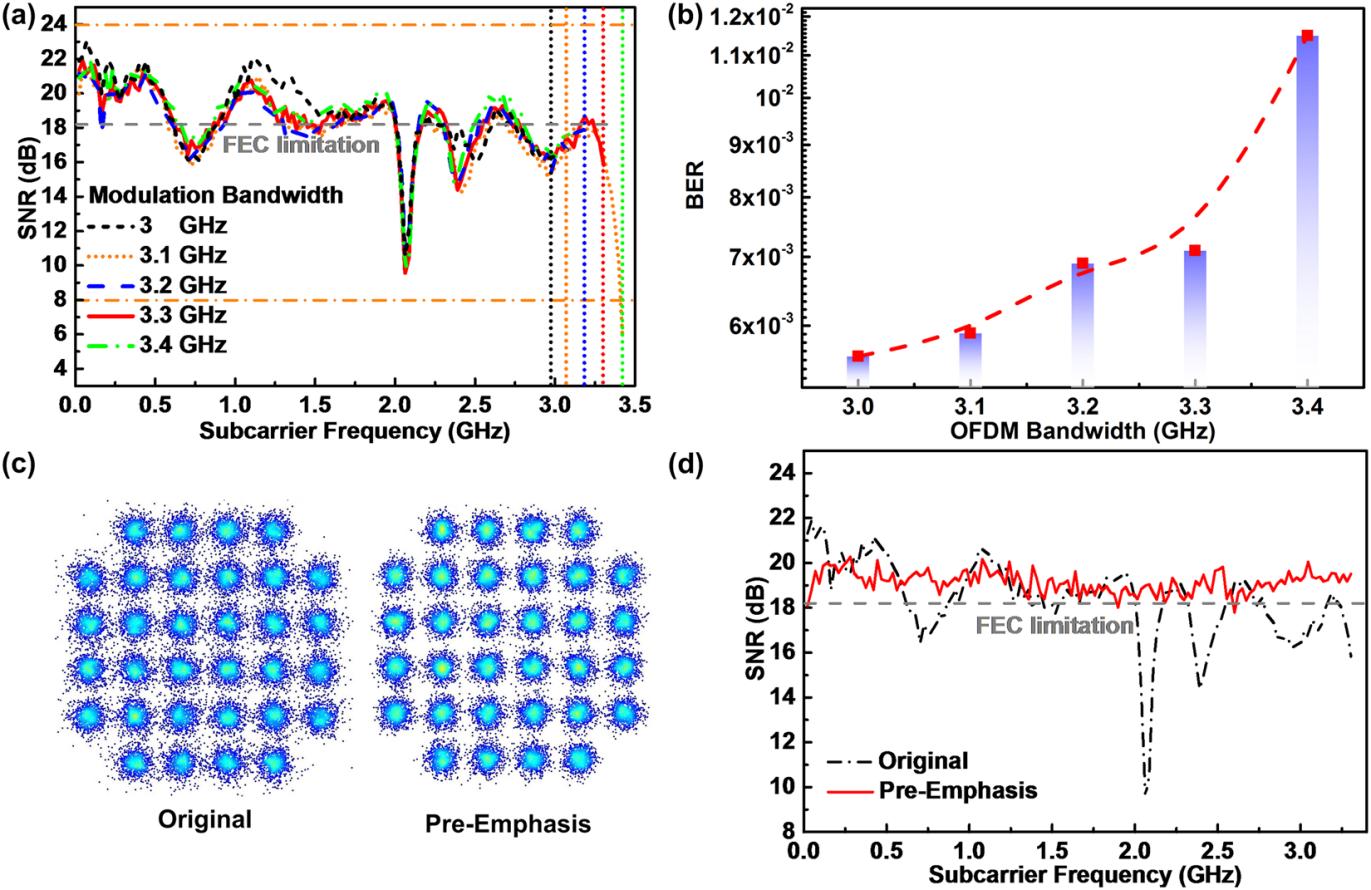
Performance of optical BtB and 3-m free-space transmitted 32-QAM OFDM data with and without pre-emphasis. (**a**) SNR and (**b**) BER performances of the 32-QAM OFDM data at different modulation data bandwidths. (**c**) Constellation plots and (**d**) SNR responses of 32-QAM OFDM data at 3.3-GHz bandwidth with and without pre-emphasis.

After optically heterodyned converting the carrier from optical to MMW domain, the SNR responses of the 32-QAM OFDM data covering different bandwidths through optical BtB and 3-m MMW transmissions are examined, as shown in Fig. 6(a). The average SNR of the received QAM-OFDM data degrades from 18.6 to 18.2 dB by enlarging the modulation bandwidth from 3 to 3.3 GHz. Further increasing the modulation bandwidth to 3.4 GHz seriously degrades the SNR by >5.8 dB at high frequencies, as the higher OFDM subcarriers near the passband edge of the bandpass filter experience more phase noise to decrease the average SNR to 17.5 dB. Figure 6(b) illustrates the corresponding BER performance to declare its degradation from 5.8 × 10^−3^ to 1.1 × 10^−2^ with expanding the modulation data bandwidth from 3 to 3.4 GHz.

**Figure 7:**
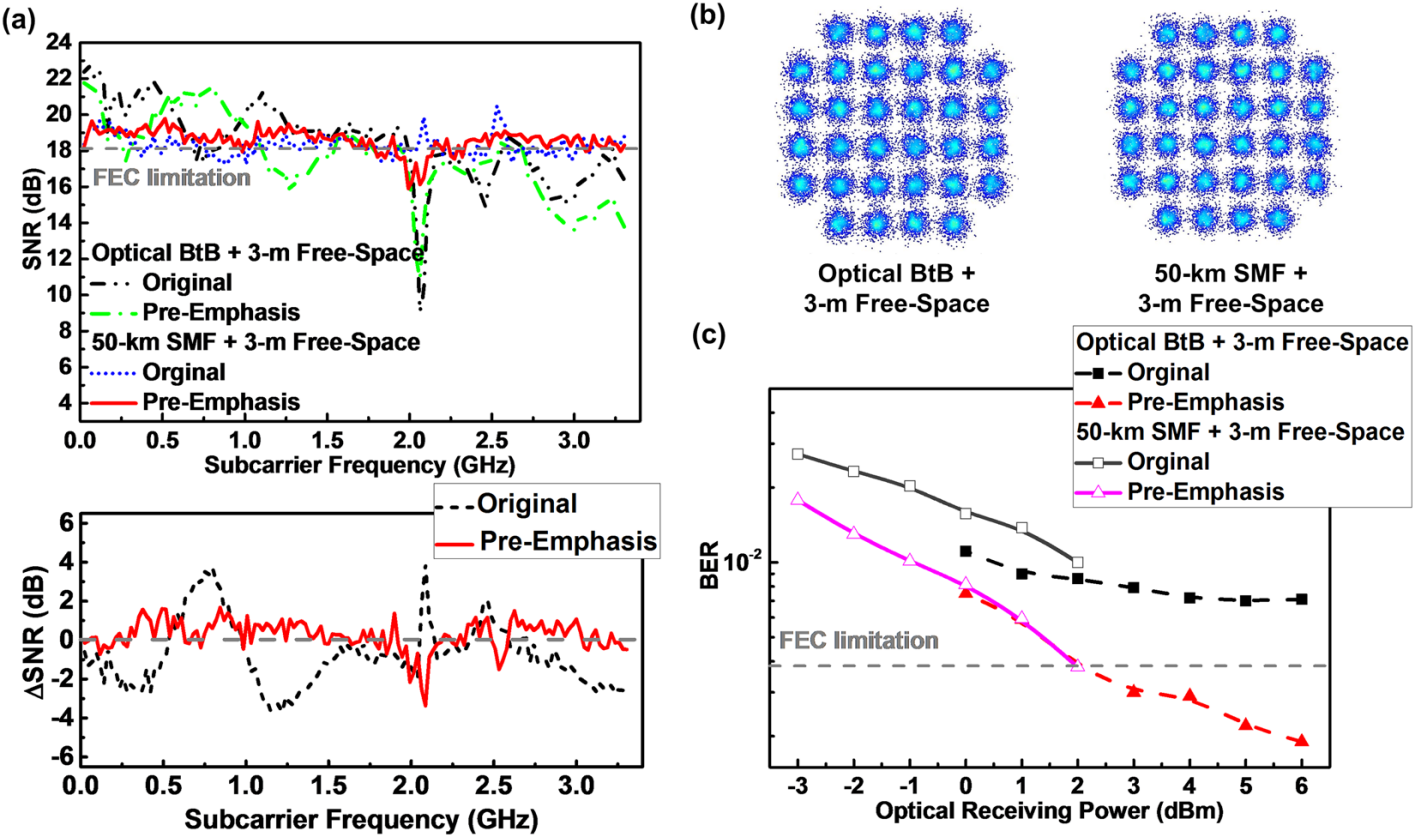
Performance of 3-m free-space transmitted 32-QAM OFDM data before and after 50-km SMF transmission with and without pre-emphasis. (**a**) SNR responses (upper) and difference (lower) of 32-QAM OFDM data at 3.3 GHz before and after 50-km SMF transmission. (**b**) Constellation plots and (**c**) BER sensitivity of 32-QAM OFDM data before and after 50-km SMF transmission.

Owing to the uneven frequency response of the used microwave components, several unwanted frequency notches occur in the SNR response to degrade the BER of the received 32-QAM OFDM data covering 3–3.4 GHz modulation bandwidth. First of all, Fig. 6(c) depicts the constellation plots of the received QAM-OFDM data with the pre-emphasis. The related EVM slightly degrades from 12.3% to 11% after implementing the pre-emphasis. Figure 6(d) compares the SNR responses within 3.3 GHz modulation data bandwidth with and without the pre-emphasis, which observes not only the >2 dB SNR improvement but also the SNR notch diminishing at 2.1 and 2.3 GHz, which optimizes the average SNR from 18.2 to 19.1 dB for reducing the BER from 7.1 × 10^−3^ to 1.9 × 10^−3^. After 50-km SMF based long-reach transmission, the upper of Fig. 7(a) shows the SNR responses of the MMW wireless 3-m free-space transmitted 32-QAM OFDM data. Unlike the optical BtB case with dual-wavelength concurrently modulated in data-stream transmission, the single-carrier modulation and transmission avoids the chromatic dispersion induced power fading on the optoelectrically generated QAM-OFDM data at 60 GHz after heterodyne detection. Therefore, the SNR degradation by the SMF propagation loss and the erbium-doped fiber amplifier (EDFA) noise is compensated by enlarging the gain of the EDFA. At an optical receiving power of 2 dBm after 50-km SMF and 3-m wireless transmissions, the SNR and BER can be respectively improved from 17.6 to 18.4 dB and 1 × 10^−2^ to 3.7 × 10^−3^ after implementing pre-emphasis, and the maximal SNR difference shown in the lower of Fig. 7(a) also shrinks from 7.5 to 4.7 dB accordingly. By attenuating the down-stream receiving power but remaining the localized TL power at 12 dBm, the receiving power sensitivity is analyzed. From the constellation plots of the 3-m free-space transmitted QAM-OFDM data with the pre-emphasis before and after 50-km SMF transmission, the EVM of the received QAM-OFDM data is only degraded by 0.1% (from 12% to 12.1%), as shown in Fig. 7(b). This is an important contribution to the fusion of MMWoF link with the long-reach PON, as the impact of SMF transmission induced RF power fading can be relieved by optical single-carrier modulation and pre-emphasis.

In optical BtB transmission, the 3-m MMW wireless transmitted 32-QAM OFDM data provides the lowest BER reducing from 7.1 × 10^−3^ to 1.9 × 10^−3^ at optical receiving power of 6 dBm with the pre-emphasis, which fails to pass the FEC if attenuating the optical receiving power to <2 dBm, as shown in Fig. 7(c). Lengthening the optical wired distance to 50 km in SMF, the lowest BER of 1 × 10^−2^ obtained at 2-dBm receiving power can be optimized to 3.8 × 10^−3^ by using the pre-emphasis. In particular, the proposed MMWoF reveals similar BER before and after 50-km SMF transmission. As long as the MMWoF link reserves sufficient optical receiving power for the QAM-OFDM data, the pre-emphasis can effectively flatten its SNR and optimize its BER to the same level.

**Figure 8:**
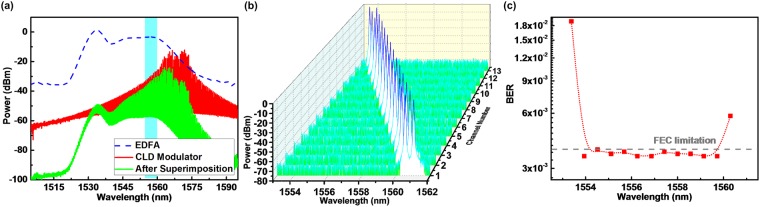
Allowable channel number of 50-km SMF and 3-m wireless transmitted 32-QAM OFDM data carried by mutually incoherent dual-wavelength optical carriers. (**a**) Optical spectra of free-running CLD modulator and long-reach boost EDFA, (**b**) optical spectra of selectable dual-wavelength DWDM channels for the proposed MMWoF, and (**c**) BER performances of 32-QAM OFDM data carried by different channels of the mutually incoherent dual-wavelength optical carriers.

To approach the multi-channel DWDM applications, the allowable channel number of the mutually incoherent dual-mode optical carriers is surveyed. Figure 8(a) illustrates the optical spectra of the free-running CLD modulator and the EDFA employed for long-reach amplification. The free-running CLD modulator reveals a central wavelength of 1571.7 nm and a wide gain spectrum ranged from 1547 to 1586 nm, which can support numerous DWDM channels for the MMWoF through the master wavelength control. To apply the proposed MMWoF for long-reach SMF transmission, the output power of the wavelength-locked CLD modulator must be sufficiently boosted to overcome the attenuation and noise. This limits the allowable channel number, as the employed EDFA has a finite and unmatched gain spectrum range (1530–1560 nm used at current stage). To declare such drawbacks, Fig. 8(a) also shows the superimposition of two gain spectra for confirming the allowable channels after optical modulation and amplification; however, the real situation of the CLD modulated optical carrier after amplification cannot be evaluated from the overlapped gain spectra as the gain competition and depletion of the EDFA at single-mode case is unclear. Therefore, only 13 MMWoF channels are selected to deliver dual-wavelength optical carriers for evaluating their transmission performances, as shown in Fig. 8(b). The BER performances of the optical 50-km SMF long-reach and MMW 3-m wireless transmitted 32-QAM OFDM data carried by different sets of mutually incoherent dual-wavelength optical carriers are compared in Fig. 8(c), which clearly shows that each available DWDM channels can successfully perform the MMWoF link at data rate of 16.5 Gbit/s individually. Note that the BER obtained in the 1^st^ and 13^th^ channels are degraded as the locked wavelength of the CLD modulator has already approached the gain spectral edge of the CLD and EDFA such that insufficient power cannot support MMWoF transmission.

During experiments, different modulation formats were encoded onto the optical baseband and the MMW passband across the different steps due to the limitation set for different carriers. At the back to back transmission, there is no dispersion effect and ASE noise from the optical amplifier. Therefore, the available modulation bandwidth is the largest among all cases, which can support up to 9-GHz wide 64-QAM OFDM at 54 Gb/s. After 50-km SMF transmission, the power fading induced by the chromatic dispersion and the ASE noise induced by the optical amplifier degrade the SNR of the delivered QAM-OFDM data. The available modulation bandwidth is decreased to 6 GHz with a data rate of 24 Gb/s. For 3-m long MMW wireless transmission, the SNR of the delivered QAM-OFDM data is seriously degraded by the wireless devices to further limit the frequency response. Hence the QAM level reduces from 64 to 32 and the available modulation bandwidth also shrinks from 6 to 3.3 GHz for supporting only 16.5 Gb/s at wireless transmission.

### Comparisons of various techniques for 60-GHz MMWoF system

To demonstrate the 60-GHz MMWoF system, different methods and related output dynamics are summarized in Table 1. Among them, mixing the baseband data with a local oscillator for frequency up-conversion before modulating onto the LD at CO is the simplest way, which allows the MMW carrier that can be remotely obtained after optical receiving at BS^39^. For confirmation, Kuri *et al*. externally modulated a distributed feedback laser diode (DFBLD) with an up-converted differential phase shift keying (DPSK) data to demonstrate a 60-GHz MMWoF system^40^. In addition, the optical single-carrier modulation is implemented to lengthen the SMF transmission distance to 85 km. To avoid the use of high-frequency mixer at the CO, optically generating the 60-GHz MMW carrier is proposed, which remotely beats the dual-mode optical carrier with optical heterodyne detection^17^. Typically, the dual-mode optical carrier can be generated by employing a CCS-DSB optical carrier, which concurrently carries the baseband data by using either an external or a direct modulator. For the externally modulated method, Weiß *et al*. used a CCS-DSB optical carrier to carry a 7.5-Gbit/s NRZ-OOK data over 50-km SMF and 36-m free space in the 60-GHz MMWoF system^23^. However, the dual-carrier modulation induces serious RF power fading effect caused by the chromatic dispersion to limit the SMF transmission distance. In 1999, Sotobayashi *et al*. employed a semiconductor optical amplifier based optical phase conjugator to reduce the chromatic dispersion induced phase difference on the DSB modulated data^41^, which lengthens the SMF transmission distance to 100 km. To provide multi-MMW-band application, Hsueh *et al*. employed a quad-mode optical carrier to simultaneously demonstrate 20- and 60-GHz MMWoF systems^42^, in which only one optical carrier carries the 2.5-Gbit/s data to achieve the single-carrier modulation for releasing the RF power fading.

**Table 1:** Optically transmitting and receiving architectures for implementing the 60-GHz MMWOF link.

Modulation	Up-Conversion	Data	Rate	Source		Distance		Advances	Ref.
				Coherent	Incoherent	Fiber	Free-Space		
External	Electrical	DPSK	0.31 Gbit/s	DFBLD		85 km	5 m	• Optical SCM	^40^
	Optical	DPSK	0.156 Gbit/s	2-λ LD		100 km	—	• Midway Optical Phase Conjugation	^41^
		OOK	7.5 Gbit/s	CCS-DSB 2-λ LD		50 km	36 m	• 2-λ	^23^
		OOK	2.5 Gbit/s	CCS-DSB 4-λ TL		50 km	4 m	• 4-λ • Optical SCM	^42^
		OFDM	28 Gbit/s	EM Laser		25 km	—	• 2-λ • Optical SCM	^28^
		OFDM	61.5 Gbit/s	EM Laser		25 km	3 m	• MIMO • Optical SCM • Bit-Loading	^43^
Direct	Electrical	OOK	0.622 Gbit/s	LD		30 km	3 m	• Optoelectronic Mixer	^44^
	Optical	OOK	5 Gbit/s	DM Laser		100 km	—	• 2-λ (External Modulation)	^47^
		BPSK	0.622 Gbit/s	2-λ FPLD		32 km	—	• 2-λ	^45^
		OFDM	6 Gbit/s	2-λ CLD		4 km	3 m	• 2-λ	^14^
		OFDM	4.3 Gbit/s	2-λ DFBLD		56 km	—	• 2-λ (Mode Deviated Locking) • Optical SCM	^32^
		OFDM	16.5 Gbit/s		Incoherent 2-λ TL	50 km	3 m	• 2-λ (Incoherent) • Optical SCM • Square-Law Down-Conversion • Pre-Emphasis • DWDM	—

Note: 2-λ: dual-mode; 4-λ: quad-mode; EM: externally modulated; DM: directly modulated; TL: tunable laser; SCM: single-carrier modulation.

To reduce the required local oscillator frequency by 2.5 times, Lin *et al*. used two dual-parallel MZMs to implement optical frequency multiplication for implementing the 60-GHz MMWoF link with the optical single-carrier modulation^28^. To further enlarge the transmission capacity, Lin *et al*. employed the 2 × 2 multi-input multi-output (MIMO) and bit-loading technics to demonstrate a 61.5-Gbit/s OFDM data transmission over 25-km SMF and 3-m free-space^43^. Although the externally modulated method can deliver the high-quality dual-mode optical carrier to demonstrate the high-speed MMWoF system, it cannot be regarded as a cost-effective and simple solution as the additional data encoder is inevitably required.

To avoid the use of the external modulation, Choi *et al*. mixed a baseband data encoded LD output with a local oscillator at the optical receiving end to demonstrate a 60-GHz MMWoF system at a cost of inevitably increasing the complexity and construction cost^44^. To solve these problems, Ogusu *et al*. employed a CCS-DSB optical master to wavelength-lock a FPLD modulator for data encoding^45^; however the injection efficiency is limited by the strong cavity effect of the FPLD. Therefore, the CLD modulator with weaker front-facet reflectance and longer cavity length is employed to provide higher injection efficiency for constructing the multi-channel MMWoF system^14^. To completely avoid the use of the CCS-DSB master for cost-effective, Chen *et al*. employed two CLD modulators to achieve master-to-slave dual-mode wavelength-locking^46^. However, the superposition of intensity noises for the master-to-slave wavelength-locked CLD inevitably degrades the carried data quality. On the other hand, Al-Dabbagh *et al*. externally modulated a directly data encoded CW laser to generate a highly-coherent dual-mode optical carrier^47^; however, simultaneously using the direct and external modulators increases the system complexity and construction cost. To approach the optical single-carrier modulation, Zhang *et al*. used a dual-mode optical master to wavelength-lock a single-mode DFBLD, which lengthens the SMF transmission distance to 56 km^32^. To further lengthen the free-space transmission distance, Tsai *et al*. employed a MMW central-carrier suppression technique with destructively interfered beating an orthogonally polarized dual-mode carrier, which ensures the MMW signal to compete more gain from the electrical amplifier^48^. With the contributions of the optical single-carrier modulation and the MMW central-carrier suppression, such an architecture significantly lengthens the SMF and free-space transmission distances to 50 km and 3 m, respectively.

For comparison, our current work employs the mutually incoherent dual-wavelength optical carrier to implement the local-oscillator-free long-reach MMWoF system at 60 GHz MMW band. The QAM-OFDM data is directly encoded onto the wavelength-locked CLD modulator based single-mode optical carrier, which can entirely acquire the optical gain during EDFA amplification for lengthening the long-reach transmission distance as compared to that carried by the dual-mode optical carrier. In addition, the optical single-mode modulation is also achieved to release the RF power fading. At the remote node, the single-mode optical carrier with data is coupled with the localized TL to form the mutually incoherent dual-wavelength optical carriers. Moreover, the square-law power envelope detection is employed to rule out the down-conversion instability caused by the MMW carrier frequency fluctuation and concurrently avoid the need of the local oscillator. By further using the pre-emphasis technique, the subcarrier SNRs of the received QAM-OFDM data can be flattened to meet the FEC criterion. This allows that the 32-QAM OFDM data at a data rate of 16.5 Gbit/s can successfully transmit over 50-km in SMF and 3-m in free-space. Furthermore, the proposed wavelength-locked CLD can simultaneously support 11 DWDM channels to enlarge the transmission capacity. These results verify that the proposed architecture with novel and simple features is very promising for the long-reach MMW fiber-wireless access link in the near future.

## Discussion

The local-oscillator-free long-reach and MMWoF link at 60 GHz is demonstrated by employing the mutually incoherent dual-wavelength laser carriers individually located at CO and remote node, which can optically heterodyned generate the 60-GHz MMW carrier to deliver the 16.5-Gbit/s 32-QAM OFDM data over 50-km in SMF and 3-m in free-space with using the pre-emphasis technique. For optical BtB transmission, the wavelength-locked CLD modulator can transmit the 64-QAM OFDM data at 54 Gbit/s with an EVM of 8%, an average SNR of 21.9 dB and a BER of 2.7 × 10^−3^ by employing the pre-emphasis technique. After transmission over 50 km in SMF, the inevitably induced RF power fading decreases the allowable transmission capacity of the QAM-OFDM data to 24 Gbit/s. At the remote node, the wavelength-locked CLD output is combined with the localized TL to form the mutually incoherent dual-wavelength optical carriers with feature of optical single-carrier modulation, which generates the optically heterodyned 60-GHz MMW carrier without suffering the RF power fading for wireless data transmission. Moreover, by using the power detector with square-law power envelope detection, the MMW carried data can be stably down-converted to the baseband with high immunity to the carrier frequency fluctuation. Finally, the mutually incoherent dual-wavelength optical carriers can successfully perform the MMWoF link at data rate of 16.5 Gbit/s in 11 DWDM channels, which verifies that the proposed architecture is very promising for the long-reach multi-channel MMW fiber-wireless access link.

## Methods

### Local-oscillator-free long-reach MMWoF system constructed with mutually incoherent dual-wavelength optical carriers at 60 GHz

**Figure 9:**
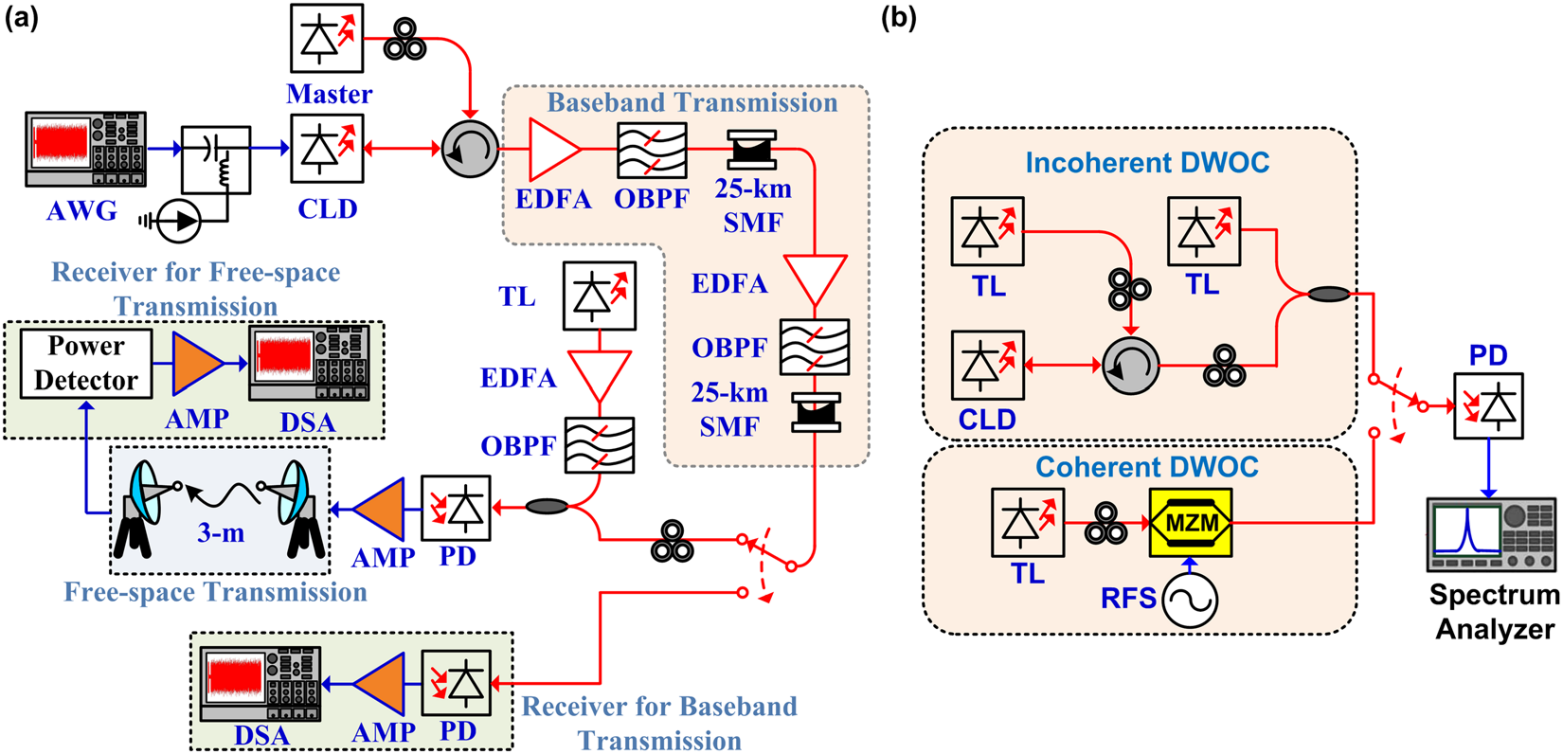
(**a**)Schematic diagram of long-reach and local-oscillator-free MMWoF system with mutually incoherent dual-wavelength optical carriers at 60 GHz. (**b**) Experimental setup for testing frequency stability of incoherently and coherently generated MMW central carriers.

The schematic diagram of the local-oscillator-free long-reach MMWoF system constructed with mutually incoherent dual-wavelength optical carriers for optical heterodyned MMW band at 60 GHz is illustrated in Fig. 9(a). A TL (HP, 8168 F) was employed as a master source for controlling the channel wavelength of the down-stream optical baseband transmission. The wavelength-lockable slave CLD modulator with a cavity length of 600 μm and a front-facet reflectance of 2% was employed as both the optical modulator and the power amplifier^49–51^, which was controlled at a temperature of 22 °C to stable its longitudinal mode frequency after master control. The CLD modulator was directly modulated with the QAM-OFDM data synthesized from the arbitrary waveform generators (AWG, Tektronix 70001A; Agilent, M8190A) at a sampling rate of 24 and 12 GS/s, respectively. As the available bandwidths of optical baseband and MMW wireless transmissions are different, the 64-QAM and 32-QAM OFDM data to be delivered over the optical wired and the MMW wireless links were individually synthesized by different AWGs (Tektronix and Agilent) covering the analog bandwidths of 15 and 5 GHz, respectively. After passing through a polarization controller and an optical circulator, the TL master with an average power of 3 dBm directly seeds into the slave CLD modulator at −3 dBm for wavelength-controlled QAM-OFDM data encoding. For long-reach optical baseband transmission over 50-km SMF, the output power of the wavelength-locked CLD modulator with the carried QAM-OFDM data was further boosted via two-stage amplification with two pairs of EDFAs, (FTTP, EDFA HA4214; Holland, NE6000L-I-2-1) and optical band-pass filters (OBPFs, Santec OTF-300; Santec OTF-910) to overcome the propagation loss. At the optical receiving end, the baseband transmitted 64-QAM OFDM data carried by the wavelength-locked CLD modulator was received by a PD (NORTEL, PP-10G) with its maximal receiving up to 11 GHz. After passing through a post-amplifier (AMP, New Focus, 1422), the received 64-QAM OFDM data was captured by a digital serial analyzer (DSA, Tektronix, DSA71604) with the analog bandwidth of 16 GHz and the sampling rate of 100 GS/s to examine its EVM, SNR, and BER performances.

For testing the MMWoF link, the down-stream optical carrier with encoded 64-QAM OFDM data was coupled with the localized free-running TL to form the mutually incoherent dual-wavelength optical carriers with optical single-carrier modulation for remotely optical heterodyned MMW carrier generation. Note that the frequency spacing between the coherence independent optical carriers was set as 60 GHz, and the optical power of the localized TL was boosted up to 12 dBm by using an EDFA-OBOF pair before optical heterodyne. After optical heterodyne with a high-speed PD, the MMW central carrier and the frequency up-mixed QAM-OFDM data at 60 GHz was generated. After passing through the electrical AMP, the 60-GHz MMW carried 64-QAM OFDM data was wirelessly delivered by using a microwave horn antenna pair through a free-space distance of 3 m. At the wireless receiving end, the MMW-carried QAM-OFDM data was down-converted into the baseband by using the square-law power envelope detector. After passing through an electrical post-amplifier, the received QAM-OFDM data was resampled by the DSA (Agilent, 91204 A) with an analog bandwidth of 12 GHz and a sampling rate of 40 GS/s to off-line analyze its transmission performances, including the EVM, SNR, and BER.

The testing bench for measuring the frequency stability of the incoherently and coherently generated MMW central carriers

Figure 9(b) illustrates the experimental setup for testing the frequency stability of the incoherently and coherently generated MMW central carriers. In the incoherent case, the free-running TL master seeds to control the CLD modulator output and the incoherent dual-wavelength optical carrier is generated via the coupling with another free-running TL output. Afterward, the MMW central carrier beating by optical heterodyne detection in the PD at 60 GHz is examined with a microwave spectrum analyzer with a resolution bandwidth of 1 kHz. In the coherence case, the free-running TL is 30-GHz modulated with a nully-biased MZM to generate a CCS-DSB optical carrier pair (an entire coherent dual-mode optical carriers), and the 60-GHz MMW carrier beating scheme remains unchanged with the incoherent case. Note that the central carrier suppression ratio of the coherently generated dual-mode optical carriers is 23.8 dB.
